# Supplementary material for: Stretchable hydrogels with low hysteresis and anti-fatigue fracture based on polyprotein cross-linkers
Source: Nat Commun. 2020 Aug 12;11:4032. doi: 10.1038/s41467-020-17877-z (PMC7423981; doi:10.1038/s41467-020-17877-z)
Supplement: Supplementary file 1 — Supplementary Information [file 41467_2020_17877_MOESM1_ESM.pdf]

Supplementary Information

Stretchable hydrogels with low hysteresis and anti-fatigue fracture based on polyprotein cross-linkers

Lei et al.

Supplementary Information

Stretchable hydrogels with low hysteresis and anti-fatigue fracture based on polyprotein cross-linkers

Hai Lei[1,2#], Liang Dong[1#], Ying Li[1,3], Junsheng Zhang[1], Huiyan Chen[1], Junhua Wu[4], Yu Zhang[1], Qiyang Fan[5,6], Bin Xue[1], Meng Qin[1], Bin Chen[5,6], Yi Cao[1,2*] and Wei Wang[1]

[1]Collaborative Innovation Center of Advanced Microstructures, National Laboratory of Solid State Microstructure, Department of Physics, Nanjing University, Nanjing, China 210093.
[2]Chemistry and Biomedicine innovation center, Nanjing University, Nanjing, China 210093.
[3]Institute of Advanced Materials and Flexible Electronics (IAMFE), School of Chemistry and Materials Science, Nanjing University of Information Science & Technology, Nanjing, China 210044.
[4]Jiangsu Key Laboratory of Molecular Medicine, Medical School, Nanjing University, Nanjing, China 210093.
[5]Department of Engineering Mechanics, Zhejiang University, Hangzhou, China 310027
[6]Key laboratory of Soft Machines and Smart Devices of Zhejiang Province, Hangzhou, China 310027

[#]equal contribution.
[*]E-mail: caoyi@nju.edu.cn.

Supplementary Figures

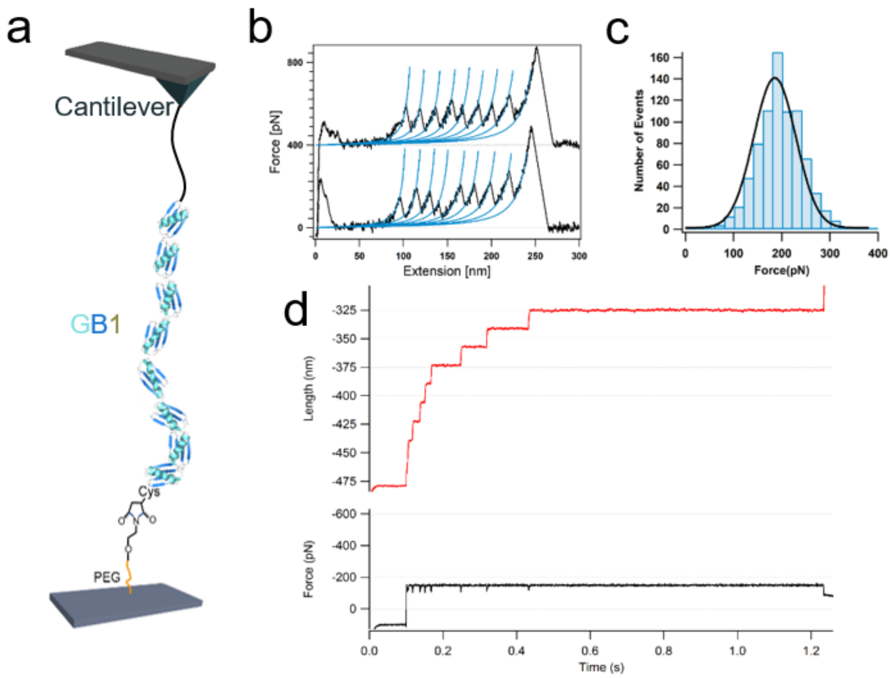

Supplementary Figure 1. Single molecule force spectroscopy (SMFS) of the polyprotein $(GB1)_8$. **a**, Schematic of the SMFS experiments performed to determine the mechanical stability of GB1. The protein was attached to the AFM cantilever through a PEG linker. **b**, Representative force-extension curves for the unfolding of $(GB1)_8$ show a saw-tooth pattern with a contour length increment of ~ 18 nm. **c**, The unfolding force distribution of GB1. **d**, Representative force-time and length-time curves of $(GB1)_8$ in the force-clamp experiment.

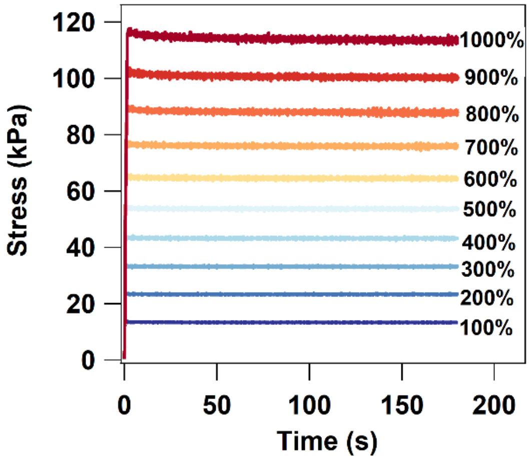

Supplementary Figure 2. Representative stress-relaxation curves of the PAA-G8 hydrogels at varying strains.

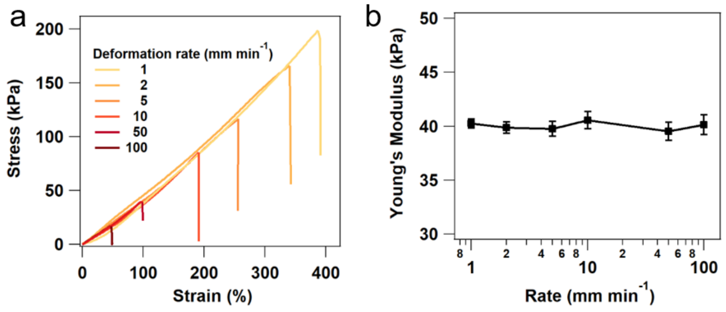

Supplementary Figure 3. Tensile test of the pure PEG hydrogel made of four-armed PEG-maleimide and dithiothreitol (DTT)**.** The hydrogel is named as the PEG-DTT hydrogel. **a**, Stress-strain curves for the hydrogel at different strain rates. **b**, Young's Modulus of the hydrogel at different strain rates. Error bars represent SD.

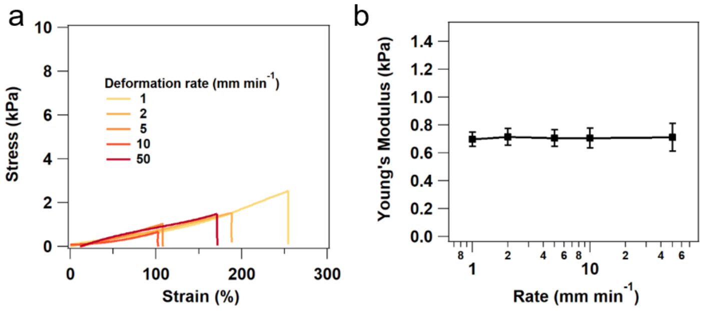

Supplementary Figure 4. Tensile test of the PEG-G8 hydrogel in the presence of 8 M urea. **a**, Stress-strain curves for the PEG-G8 hydrogel with urea at different strain rates. **b**, Young's Modulus of the PEG-G8 hydrogel with urea at different strain rates. Error bars represent SD.

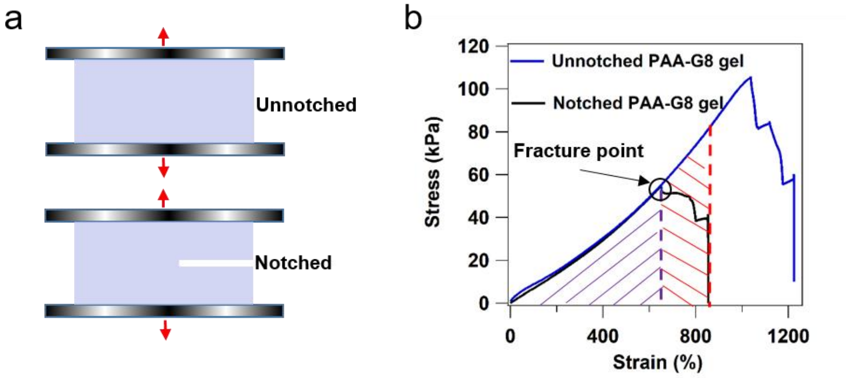

Supplementary Figure 5. Method to measure hydrogel toughness. **a**, Stretching two samples of the same material, one containing no crack and the other containing a precut crack. **b**, The stress-strain curves of the two samples. The hydrogel toughness is calculated by the shaded area multiply by the original length of the hydrogel.

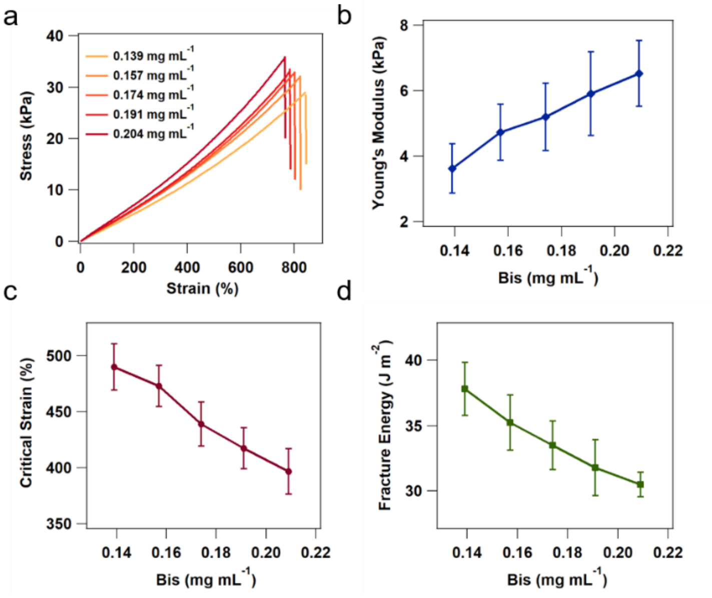

Supplementary Figure 6. Compositions greatly affect the behavior of the PAA hydrogel. **a**, Stress-strain curves of gels of various concentrations of bisacrylamide (Bis) but the same acrylamide (100 mg mL^{-1}), as labelled. Each test was conducted by pulling an unnotched sample to rupture. **b**, Elastic moduli calculated from stress-strain curves, plotted against Bis concentration. **c**, Critical strain, for notched gels of various concentrations of Bis, measured by pulling the gels to rupture. **d**, Fracture energy, as a function of concentrations of Bis. Error bars represent SD.

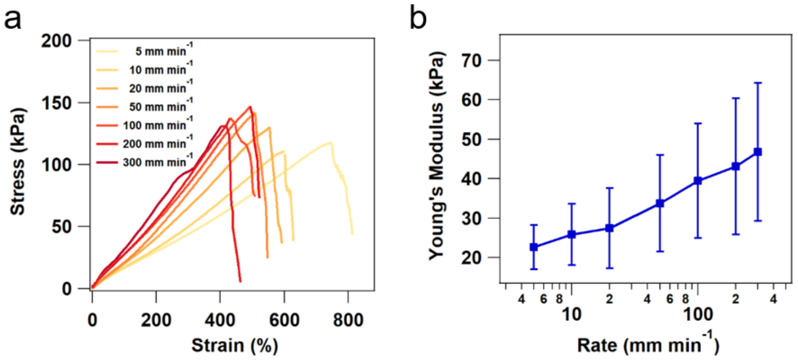

Supplementary Figure 7. Tensile test of the PAA-G8 hydrogels (120 mg mL^{-1}). **a**, Stress-strain curves for the hydrogels at different strain rates. **b**, Young's Modulus of the hydrogels at different strain rates. Error bars represent SD.

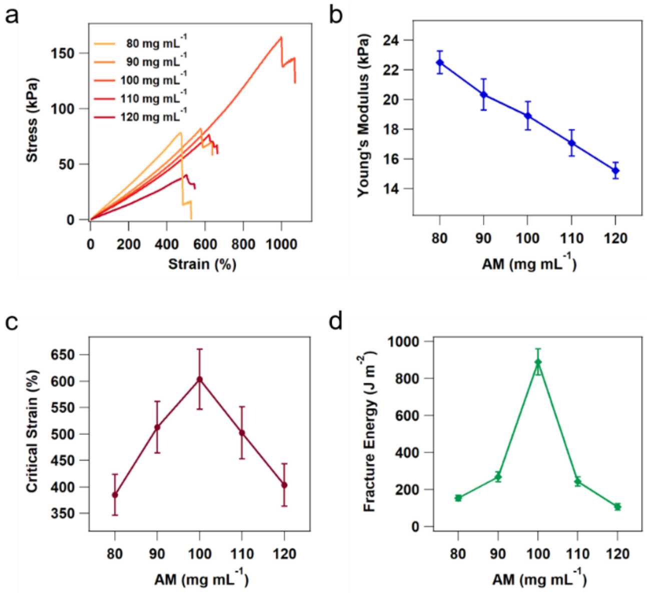

Supplementary Figure 8. Compositions greatly affect the behavior of the PAA-G8 hydrogel. **a**, Stress-strain curves of gels of various concentrations of acrylamide (AM) but the same SNAP-G8-SNAP (100 mg mL^{-1}), as labelled. Each test was conducted by pulling an unnotched sample to rupture. **b**, Elastic moduli calculated from stress-strain curves, plotted against acrylamide concentration. **c**, Critical strain, for notched gels of various concentrations of acrylamide, measured by pulling the gels to rupture. **d**, Fracture energy, as a function of concentrations of acrylamide. Error bars represent SD.

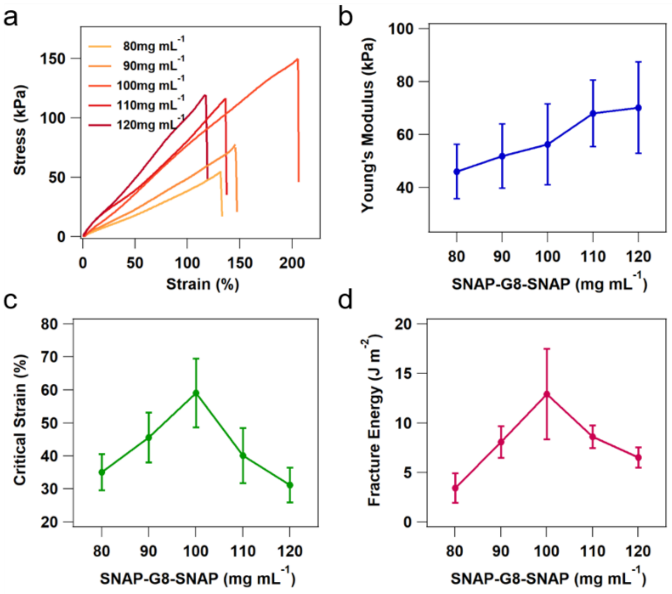

Supplementary Figure 9. Composition greatly affects the mechanical behaviors of the PEG-G8 hydrogel. **a, b,** Stress-strain curves and the corresponding elastic moduli of the hydrogels of various concentrations of SNAP-G8-SNAP and the same 4-armed-PEG-SH. The samples were unnotched. **c, d,** Critical strain and fracture energy of notched hydrogels of various concentrations of SNAP-G8-SNAP. Error bars represent SD.

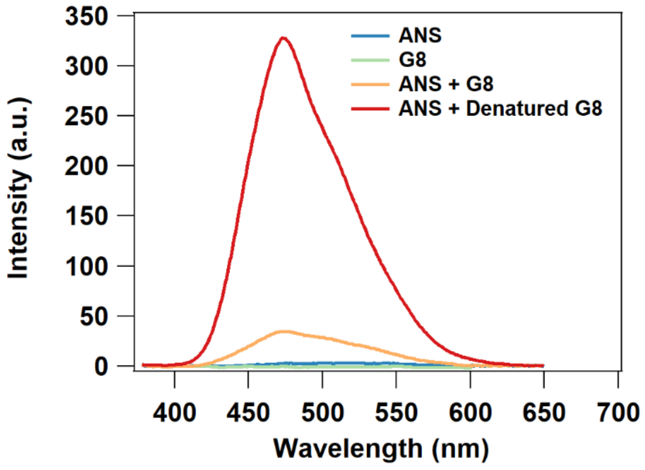

Supplementary Figure 10. The fluorescence intensity at 480 nm for ANS, polyprotein G8, the mixture of ANS/G8 and mixture of ANS/denatured G8.

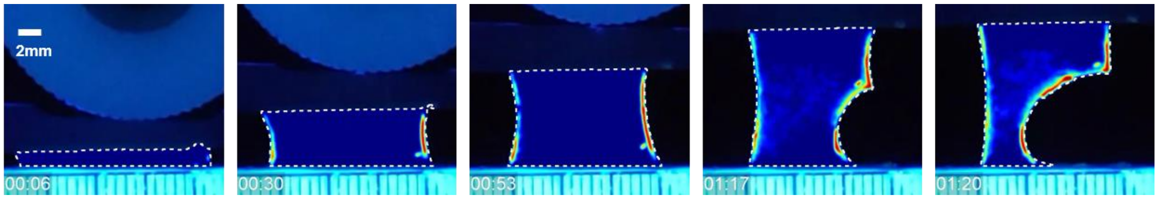

Supplementary Figure 11. Sequential images of stretching an intact PAA-G8 hydrogel (G8: 100 mg mL^{-1}) in the presence of an environment sensitive dye, ANS.

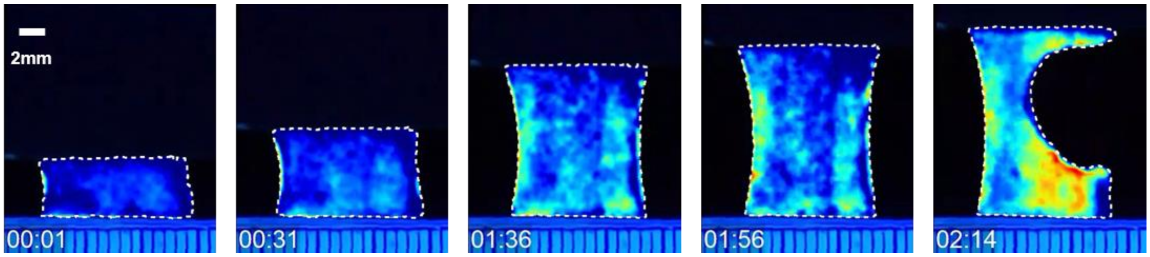

Supplementary Figure 12. Sequential images of stretching an intact PEG-G8 hydrogel in the presence of an environment sensitive dye, ANS. The fluorescence of ANS became brighter when it bound with the hydrophobic residues upon GB1 unfolding.

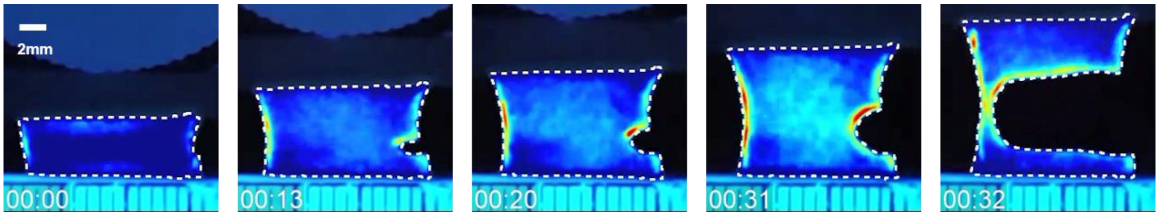

Supplementary Figure 13. Sequential images of stretching a notched PEG-G8 hydrogel in the presence of an environment sensitive dye, ANS.

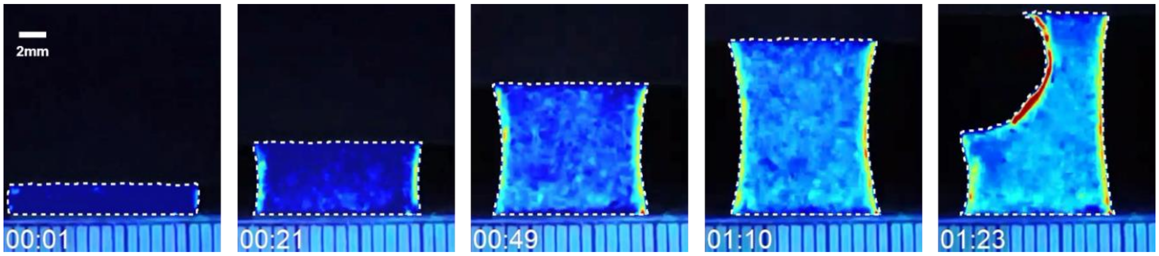

Supplementary Figure 14. Sequential images of stretching an intact PAA-G8 hydrogel (G8: 120 mg mL^{-1}) in the presence of an environment sensitive dye, ANS. In this hydrogel, the crack started to propagate from the left side, presumably due to the presence of defects there. (Supplementary Movie 5)

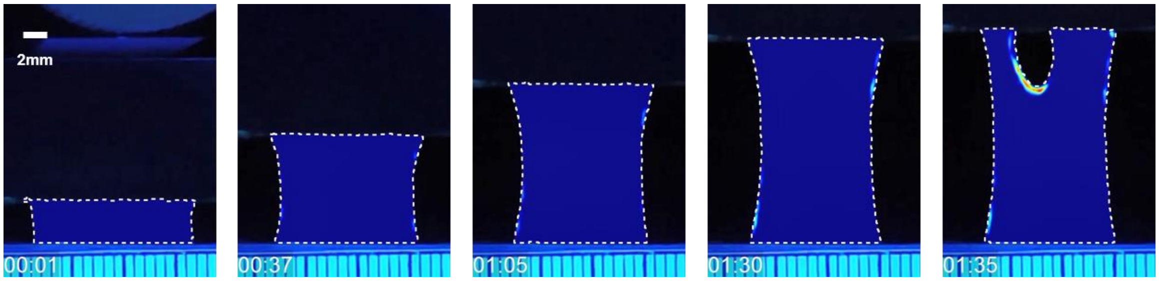

Supplementary Figure 15. Sequential images of stretching an intact PAA-G8 hydrogel (G8: 80 mg mL^{-1}) in the presence of an environment sensitive dye, ANS. In this hydrogel, the crack started to propagate from the top side, presumably due to the presence of defects there. (Supplementary Movie 6)

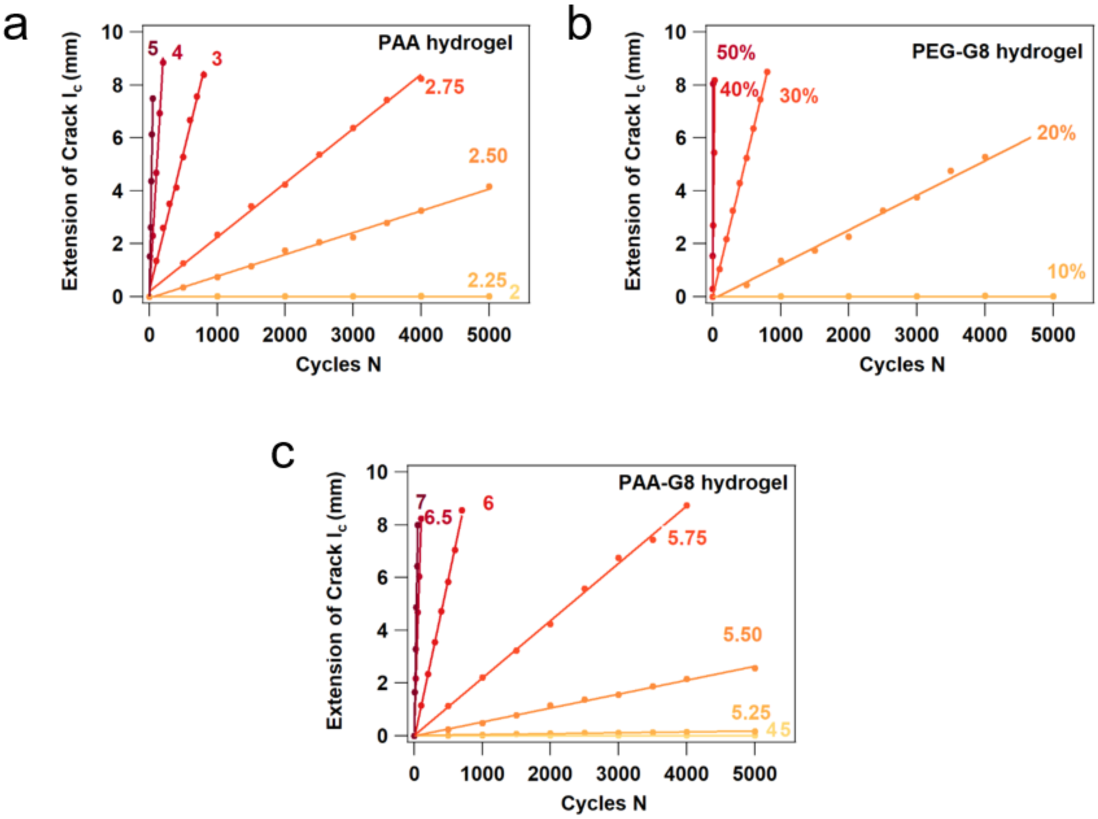

Supplementary Figure 16. Fatigue threshold calculation. Crack extension as a function of the number of cycles with different applied strain for PAA hydrogel (**a**), PEG-G8 hydrogel (**b**) and PAA-G8 hydrogel (**c**), respectively.

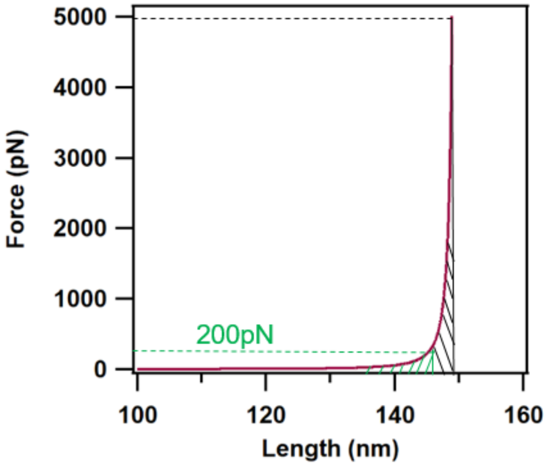

Supplementary Figure 17 Simulation of stretching a polymer to different forces using Worm-Like-Chain model (contour length = 150 nm and persistence length = 0.4 nm). The shaded areas represent the stored potential energy under specific forces.

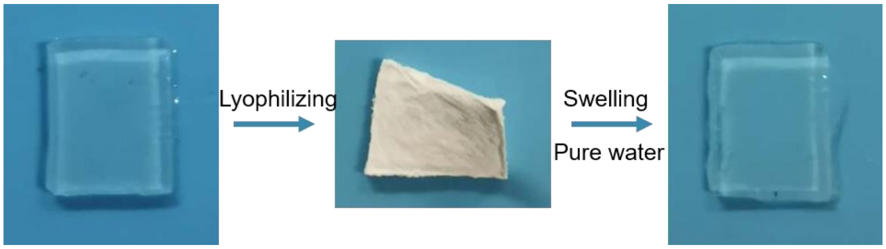

Supplementary Figure 18. Photos of the hydrogel swollen in pure water (left), after drying (middle) and reswelling (right).

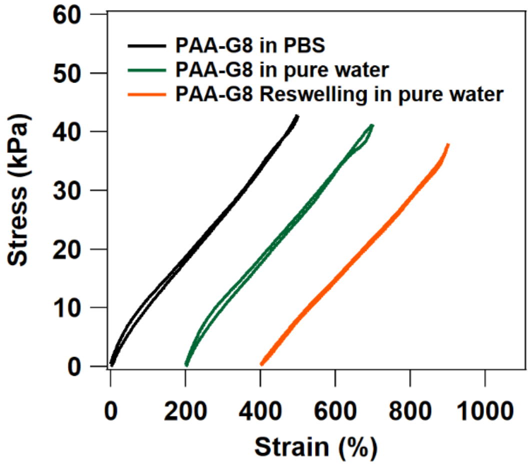

Supplementary Figure 19. The stress-strain curves for the PAA-G8 hydrogel in PBS (black line), pure water (green line) and reswelling in pure water (orange line). The curves were horizontally offset for clarity.

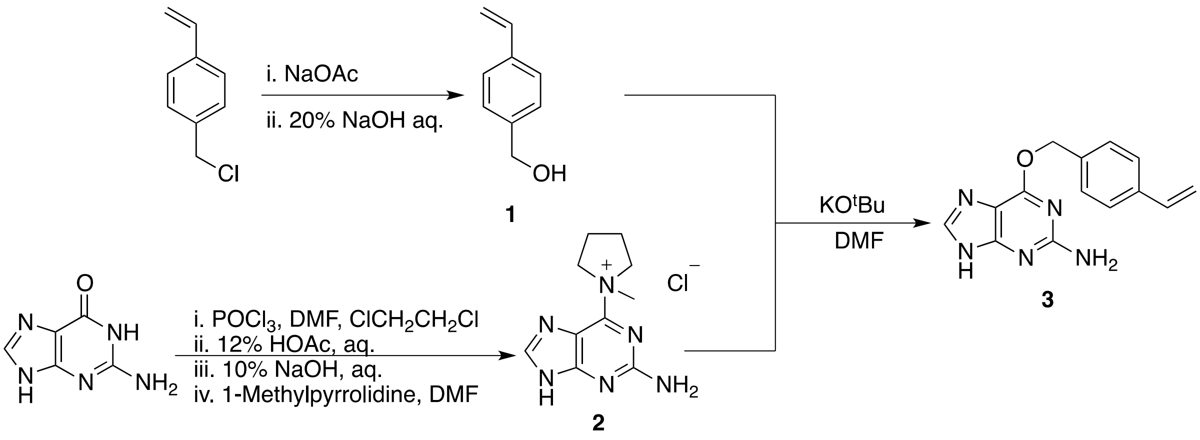

Supplementary Figure 20. The outline of the synthesis of BS. It includes three steps.

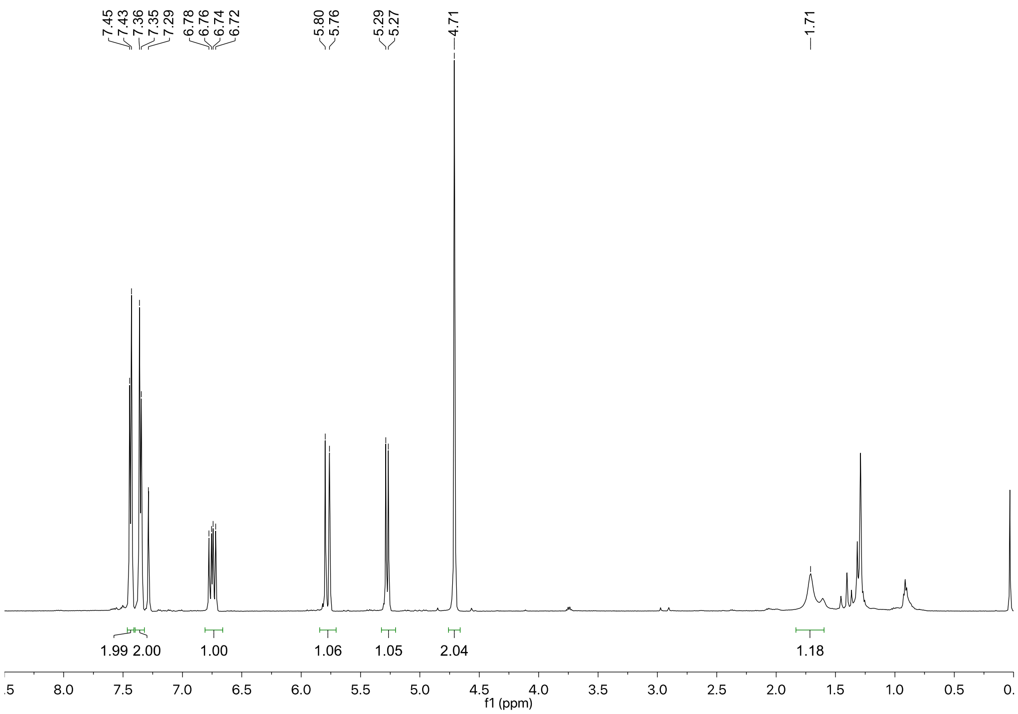

Supplementary Figure 21. The NMR of the 4-vinylbenzyl alcohol.

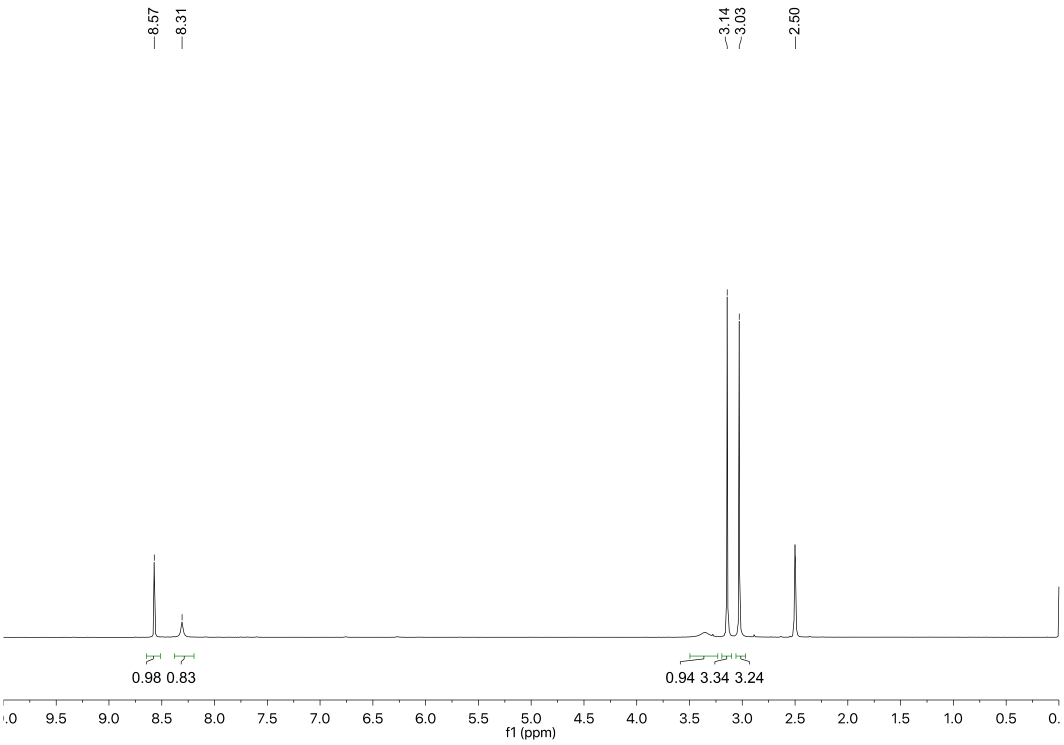

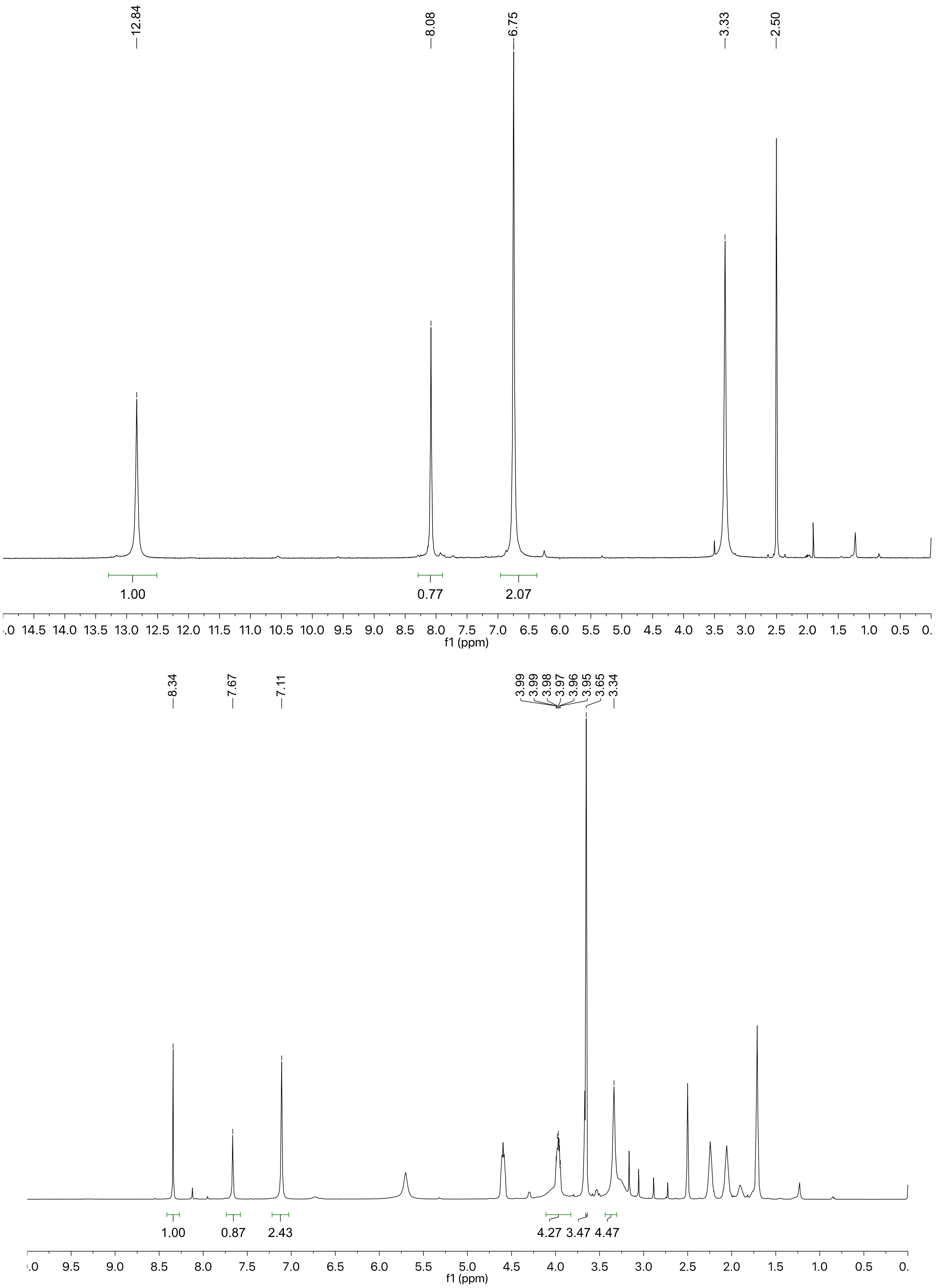

Supplementary Figure 22. The ^{1}H NMR spectra of the intermediate **A**, intermediate **B** and product **2**, respectively.

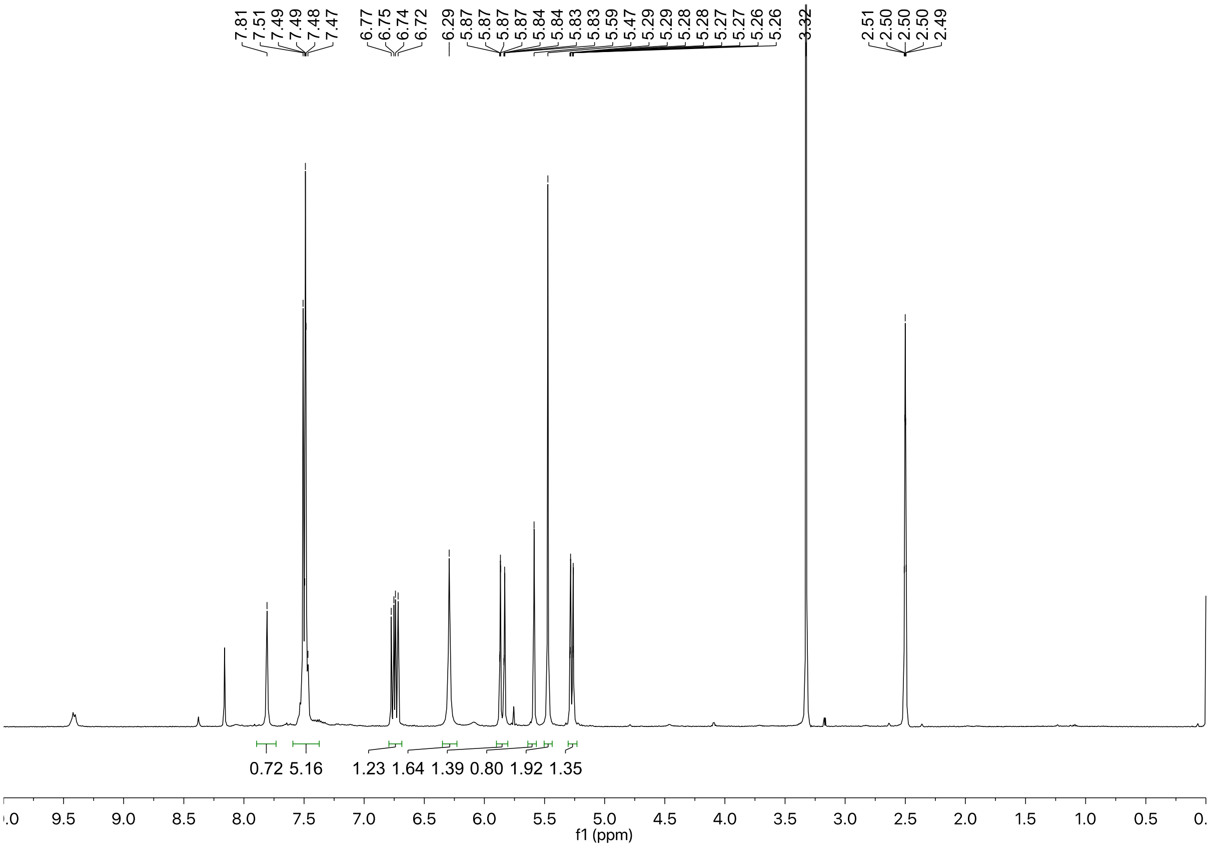

Supplementary Figure 23. The NMR of the **BS**.

Supplementary Methods

Single molecule force spectroscopy

Single molecule AFM experiments were carried out on a commercial AFM (ForceRobot 300, JPK, Berlin, Germany) in PBS buffer at room temperature. The spring constants of the AFM cantilevers (MLCT from Bruker) were calibrated using the equipartition theorem before each experiment, with typical values of 50 pN nm^{-1}. The pulling speeds were 1600 nm s^{-1} for force extension experiment and the force clamp experiment under a constant force of 150 pN.

Measurement of the energy release rate

The extension of crack per cycle, *dc/dN*, is measured from the images of the undeformed hydrogels before and after the Nth cycle. $dc/dN = L_N - L_{N-1}$, where L_N and L_{N-1} are the length of the crack after the Nth and $(N-1)^{th}$ stretching-relaxation cycle with strain λ, respectively.

The energy release rate G of the notched hydrogel takes the following form:

$$G = HW(\lambda_{max}), \quad (1)$$

Where H is the distance between the two grippers of the tensile tester when the notched hydrogel is undeformed; $W(\lambda_{max})$ is the energy per volume of the uncut sample while stretched; and λ_{max} is the critical strain in which the crack growth rate is the lowest obtained in experiments. The energy density $W(\lambda_{max})$ is obtained by integrating the area below the stress-strain curve of the unnotched sample.

Estimating the fatigue threshold using the Lake-Thomas model

In the Lake-Thomas theory, the fracture threshold is equal to the number of chains per cross-sectional area multiplied by the energy that is required to break one bridging strand[1],

$$\Gamma = \sigma W = \frac{1}{2} \upsilon_0 R_0 n U, \quad (2)$$

where R_0 is the average end-to-end distance of an elastically active network strand in its undeformed state, υ_0 is the number density of such elastically active subchains, n is the average number of repeat units along the bridging strand, and U is the energy that is stored in each repeat unit when the bridging strand breaks. The prefactor of 1/2 comes from the projection of the end-to-end vectors of subchains onto the normal of the crack plane.

Suo and colleagues have recently applied the Lake-Thomas theory to calculated the theoretical fatigue threshold of single-network PAA hydrogels as follows[2]:

$$\Gamma_0 = \phi_{PAA}^{2/3} bUln^{1/2}, \quad (3)$$

where ϕ_{PAA} is the volume fraction of the polyacrylamide network in the PAA hydrogels, b is the number of bonds per unit volume of the dry polymer, U is the C-C bond energy, l is the length of each monomer unit and n is the number of monomer units in a polyacrylamide chain. For PAA hydrogel, the volume fraction of the polyacrylamide network can be estimated by

$$\emptyset_{PAA} = \frac{(1-w_{water})\times\frac{m_{PAA}}{m_{PAA}+m_{Bis}}}{\rho_{AAM}} \times \rho_{gel}. \quad (4)$$

The density of acrylamide ρ_{AAM} is 1.1g cm^{-3}. The density of the hydrogel is approximately 1 g cm^{-3}. The

volume fraction of the polyacrylamide network in the hydrogel is 5.68 vol%. The number of bonds per unit of the dry polymer is estimated by the number of monomers per volume of the dry polymer, $b=A\rho/M=9.32\times10^{27}$ m^{-3}, where A is the Avogadro number (6.022×10^{23}) and M is the molecular weight of acrylamide (71.08 g mol^{-1}). The energy of a C-C bond U is 3.3×10^{-19} J. The length of the monomer is estimated by $l=b^{-1/3}=0.475$ nm. The molar ratio of the cross-linker bisacrylamide relative to the monomer acrylamide is 0.079%. One cross-link connects two polymer chains, so the number of monomer between two cross-links n is estimated by $n = 1/(2\times0.079\%) = 633$. Accordingly, the fatigue thresholds of PAA hydrogel is predicted to be 5.1 J m^{-2}.

In the PAA-G8 hydrogel, we revised the Lake-Thomas theory to consider the energy dissipation from GB1 unfolding. Every GB1 unfolding event leads to a release of potential energy stored by the bridging strand and a change of the end-to-end distance of the network strand. The final energy release comes from the rupture of the bridging strain. Considering the bridging strain made of the PAA chain and eight GB1 domains, the crack energy threshold can be estimated as follows:

$$\Gamma = \sum_{\delta=0}^{7} \frac{1}{2} \upsilon_0 R (n_a + 3\delta N_{GB1}) U_{uf} + \frac{1}{2} \upsilon_0 (R' + 8L_0)(n_a + 3\delta N_{GB1}) U_{break}$$

$$R = \begin{cases} R_0, \delta = 0, \\ R' + \delta L_0, \delta \neq 0, \end{cases} \tag{5}$$

where υ_0 the number density, R is the average end-to-end distance of the active subchains, n_a is the number of acrylamide per unit, δ is the number of unfolded GB1, N_{GB1} represents the number of amino acids of GB1, R_0 is the average end-to-end distance of the active subchains in initial, R' is the end-to-end length of the bridge strand before one of GB1 unfolding, U_{uf} is the energy strored in C-C bond at the unfolding force of GB1 and U_{break} is the energy to break C-C bond.

In this equation, the first part corresponds to the energy release due to the unfolding of GB1 domains and the second part correspond to the energy release due to the rupture of the polymer network after the unfolding of all GB1 domains. As the initial concentration of SNAP-G8-SNAP in our hydrogel is 100 mg mL^{-1}, and its molecular weight is 8.9×10^{4} g mol^{-1}, with the swelling ratio of 1.6, υ_0 is estimated to be 4.2×10^{23} m^{-3} and R_0 is 28 nm. The number of acrylamide per unit n_a is 1266. U_{uf} is the energy stored in C-C bond at the unfolding force of GB1 (typically 200 pN). Based on the simulation using Worm-Like-Chain model, the potential energy under 200 pN is about 30% of the energy to break a C-C bond ($U_{break}=3.3\times10^{-19}$ J), showed in Supplementary Figure 17. Thus U_{uf} is about 0.99×10^{-19} J per bond. R' is the end-to-end length of the bridge strand before one of GB1 unfolding, which is estimated to be 152 nm (the length per bond 0.12nm multiply n_a). N_{GB1} =60 is the number of amino acids of GB1 and L_0 =21.8 nm is the contour length of GB1. Therefore, the crack energy threshold is estimated to be 138 J m^{-2}.

Synthesis of O6-benzylguanine styrene (BS)

Synthesis of 4-vinylbenzyl alcohol (**1**)[3]

4-Vinylbenzyl chloride (2.05 mL, 14.55 mmol) and sodium acetate (1.29 g, 15.73 mmol) in DMSO (6 mL) was incubated and stirred at 45 ^oC under nitrogen atmosphere for 24 hr. The reaction was cooled to room temperature and poured to water (10 mL). The aqueous mixture is extracted with three 10-mL ethyl acetate. The organic layers were combined and dried over anhydrous sodium sulfate. The solid was filtered off and the filtrate was concentrated in vacuo. The residue in ethanol (5 mL) was directly added with 20% aqueous sodium hydroxide solution (5 mL) and the mixture was heated to reflux for 4 hr. The reaction was then cooled to room temperature and extracted with three 50-mL ethyl acetate. The organic layers were combined, washed with brine (50 mL), and dried over anhydrous sodium sulfate. The solid was filtered off and the volatile was removed to give pale brown oil, which was further dried in vacuum to afford the desired product (1.95 g, yield 99.9 %). ^1H NMR(500 MHz, $CDCl_3$): δ *(ppm)* 7.44 (d, 2 H, *J*= 7.89 Hz), 7.35 (d, 2 H, *J*= 7.88 Hz), 6.75 (dd, 2 H, J_1= 17.56 Hz, J_2= 10.88 Hz), 5.78 (d, 1 H, *J*= 17.59 Hz), 5.28 (d, 1 H, *J*= 10.85 Hz), 4.71 (s, 2 H), 1.71 (br, 1 H), showed in Supplementary Figure 21.

Synthesis of compound (**2**)[4,5]

Guanidine (4.53 g, 30 mmol) and dimethylformamide (14 mL) in 1,2-dichloroethane (50 mL) was added with $POCl_3$ (8.4 mL, 90 mmol) and the resulting mixture was heated to 80 ^oC for 8 hr. The reaction was cooled to room temperature and slowly poured into water (120 mL). Sodium carbonate (~18 g) was added in small portions to neutralize the mixture to pH ~ 5. The mixture is then transferred to a separatory funnel to allow layers to separate. The aqueous layer was collected and sodium hydroxide (2.52 g) was slowly added to the aqueous solution, during which yellow-brown precipitate appeared. The mixture was centrifuged and the solid was further washed with water (100 mL) and then dried over vacuum.[6] Yellow solid (intermdediate **A**, 4.72 g, yield 65.4%) was used directly without further purification. ^1H NMR (500 MHz, d_6-DMSO): δ *(ppm)* 8.57 (s, 1 H), 8.31 (s, 1 H), 3.34 (s, 3 H) 3.14 (s, 3 H), 3.03 (s, 3 H).

All solid from the previous step (4.72 g, 19.6 mmol) was added to 12% acetic acid (56 mL) and the resulting mixture was stirred at 70 ^oC for 4.5 h. The reaction was cooled to room temperature and centrifuged to collect the solid. The solid was further washed with water until the aqueous supernatant was no longer acidic and dried over high vacuum to give brown solid as the product (3.32 g, yield 85.7%). The solid was directly treated with 30 mL 10% sodium hydroxide at room temperature for 3 h. The mixture was neutralized with concentrated hydrochloric acid to pH 5~7. The solid was collected by centrifugation and dried over high vacuum to afford yellow solid[6] (intermediate **B**, 2.55 g, 89.3%). ^1H NMR (500 MHz, d_6-DMSO): δ *(ppm)* 12.84 (s, 1 H), 8.06 (s, 1 H), 6.75 (s, 2 H).

The yellow solid (2.55 g,15 mmol) in the previous step was suspended in *N*, *N*-dimethyl formamide (90 mL) and heated to 60 ^oC. When all was dissolved, the solution was cooled to room temperature and 1-methylpyrrolidine (3.4 mL, 32.7 mmol) was added and the resulting mixture was stirred at room temperature for 32 h. Then acetone (5 mL) was added to precipitate the solution. The solid was collected by filtration, washed with diethyl ether (50 mL), and dried over high vacuum to afford the desired product **2** as a whitish powder (2.05 g, yield 53.4%; The yield over 4 steps was 26.8%). ^1H NMR (500

MHz, d_6-DMSO): δ *(ppm)* 8.34 (s, 1 H), 7.67 (s, 1 H), 7.11 (s, 2 H), 3.98 (m, 4 H), 3.65 (s, 3 H), 3.34 (s, 4 H), showed in Supplementary Figure 22.

Synthesis of **BS** (**3**)

4-Vinylbenzyl alcohol (**1**, 136 mg, 1.02 mmol) was dissolved in anhydrous *N*, *N*-dimethylformamide (10 mL) and potassium *tert*-butoxide (246 mg, 2.19 mmol) was added to the solution. After the resulting mixture was stirred at room temperature over 5 min., compound **2** (123 mg, 0.48 mmol) was added in one portion to the slurry and the reaction was stirred at room temperature for 3.5 h. The reaction mixture was filtered and the filtrate was concentrated in vacuum. The residue was subjected to flash chromatography for further purification using the gradient mobile eluent from 1% to 6% methanol in dichloromethane to give pale yellow solid as the desired product (63.5 mg, yield: 23.4%). ^{1}H NMR (500 MHz, d_6-DMSO): δ *(ppm)* 7.81 (s, 1 H), 7.50 (m, 5 H), 6.74 (dd, 1 H, J_1=17.65 Hz, J_1=10.95Hz) 6.29 (s, 2 H),5.85 (m, 1 H), 5.59 (s, 1 H), 5.47 (s, 2 H), 5.27 (m, 1 H); ESI: calcd. for $C_{14}H_{14}N_5O^+$ 268.12, found: 268.10, showed in Supplementary Figure 23.

Supplementary Reference

1 Shu, W. et al. Quantitative Adjustment to the molecular Energy Parameter in the Lake-Thomas Theory of Polymer Fracture Energy. *Macromolecules* **52**, 2772-2777 (2019).

2 Zhang, W. *et al*. Fracture Toughness and Fatigue Threshold of Tough Hydrogels. *ACS Macro Lett.* **8**, 17-23 (2019).

3 Alexander, W. *et al.* The formation of core cross-linked star polymer and nanogel assemblies facilitated by the formation of dynamic covalent imine bonds. *Poly. Chem.* **2**, 2500 (2011).

4 Antje, K. *et al*. A general method for the covalent labeling of fusion proteins with small molecules in vivo. *Nat. Biotechnol.* **21**, 86-89 (2003).

5 Tomonori, K. *et. al.* Highly Activatable and Environment-Insensitive Optical Highlighters for Selective Spatiotemporal Imaging of Target Proteins. *J. Am. Chem. Soc.* **134**, 11153-11160 (2012).

6 Mikhail, Y. *et. al.* Facile Synthesis of 8-Azido-6-Benzylaminopurine. *Nucleosides, Nucleotides & Nucleic Acids*, **30**, 503-511 (2011).
