# Supplementary material for: Stretchable hydrogels with low hysteresis and anti-fatigue fracture based on polyprotein cross-linkers
Source: Nat Commun. 2020 Aug 12;11:4032. doi: 10.1038/s41467-020-17877-z (PMC7423981; doi:10.1038/s41467-020-17877-z)
Supplement: Supplementary file 3 — Description of Additional Supplementary Files [file 41467_2020_17877_MOESM3_ESM.pdf]

Description of Additional Supplementary Files

File Name: Supplementary Movie 1
Description: Video of stretching a noteched PAA-G8 hydrogel (G8: 100 mg mL^{-1}) in the presence of an environment sensitive dye, ANS.

File Name: Supplementary Movie 2
Description: Video of stretching an intact PAA-G8 hydrogel (G8: 100 mg mL^{-1}) in the presence of an environment sensitive dye, ANS.

File Name: Supplementary Movie 3
Description: Video of stretching an intact PEG-G8 hydrogel in the presence of an environment sensitive dye, ANS.

File Name: Supplementary Movie 4
Description: Description: Video of stretching a notched PEG-G8 hydrogel in the presence of an environment sensitive dye, ANS.

File Name: Supplementary Movie 5
Description: Video of stretching an intact PAA-G8 hydrogel (G8: 120 mg mL^{-1}) in the presence of an environment sensitive dye, ANS.

File Name: Supplementary Movie 6
Description: Video of stretching an intact PAA-G8 hydrogel (G8: 80 mg mL^{-1}) in the presence of an environment sensitive dye, ANS.
